# Galectin‐9 and CXCL10 as Biomarkers for Disease Activity in Juvenile Dermatomyositis: A Longitudinal Cohort Study and Multicohort Validation

**DOI:** 10.1002/art.40881

**Published:** 2019-03-12

**Authors:** Judith Wienke, Felicitas Bellutti Enders, Johan Lim, Jorre S. Mertens, Luuk L. van den Hoogen, Camiel A. Wijngaarde, Joo Guan Yeo, Alain Meyer, Henny G. Otten, Ruth D. E. Fritsch‐Stork, Sylvia S. M. Kamphuis, Esther P. A. H. Hoppenreijs, Wineke Armbrust, J. Merlijn van den Berg, Petra C. E. Hissink Muller, Janneke Tekstra, Jessica E. Hoogendijk, Claire T. Deakin, Wilco de Jager, Joël A. G. van Roon, W. Ludo van der Pol, Kiran Nistala, Clarissa Pilkington, Marianne de Visser, Thaschawee Arkachaisri, Timothy R. D. J. Radstake, Anneke J. van der Kooi, Stefan Nierkens, Lucy R. Wedderburn, Annet van Royen‐Kerkhof, Femke van Wijk

**Affiliations:** ^1^ University Medical Centre Utrecht Utrecht The Netherlands; ^2^ Beatrix Children’s Hospital University Medical Centre Groningen Groningen The Netherlands; ^3^ Emma Children’s Hospital AMC University of Amsterdam The Netherlands; ^4^ Sophia Children’s Hospital, Erasmus University Medical Centre, Rotterdam, The Netherlands, and Leiden University Medical Centre Leiden The Netherlands; ^5^ University College London, University College London Hospital, the NIHR Biomedical Research Centre at Great Ormond Street Hospital, and Great Ormond Street Hospital London UK; ^6^ University College London London UK; ^7^ Lausanne University Hospital, Lausanne, Switzerland, and University Hospital Basel Basel Switzerland; ^8^ Academic Medical Centre Amsterdam Amsterdam The Netherlands; ^9^ University Medical Centre Utrecht, Utrecht, The Netherlands, and Radboud University Medical Centre Nijmegen The Netherlands; ^10^ KK Women’s and Children’s Hospital, Duke‐NUS Medical School SingHealth Duke‐NUS Academic Medical Center Singapore; ^11^ CHU de Strasbourg Strasbourg France; ^12^ University Medical Centre Utrecht, Utrecht, The Netherlands, Sigmund Freud Private University, Vienna, Austria, and Hanusch Krankenhaus und Ludwig Boltzmann Institut für Osteologie Vienna Austria; ^13^ Sophia Children’s Hospital Erasmus University Medical Centre Rotterdam The Netherlands; ^14^ Amalia Children’s Hospital Radboud University Medical Centre Nijmegen The Netherlands

## Abstract

**Objective:**

Objective evaluation of disease activity is challenging in patients with juvenile dermatomyositis (DM) due to a lack of reliable biomarkers, but it is crucial to avoid both under‐ and overtreatment of patients. Recently, we identified 2 proteins, galectin‐9 and CXCL10, whose levels are highly correlated with the extent of juvenile DM disease activity. This study was undertaken to validate galectin‐9 and CXCL10 as biomarkers for disease activity in juvenile DM, and to assess their disease specificity and potency in predicting the occurrence of flares.

**Methods:**

Levels of galectin‐9 and CXCL10 were measured by multiplex immunoassay in serum samples from 125 unique patients with juvenile DM in 3 international cross‐sectional cohorts and a local longitudinal cohort. The disease specificity of both proteins was examined in 50 adult patients with DM or nonspecific myositis (NSM) and 61 patients with other systemic autoimmune diseases.

**Results:**

Both cross‐sectionally and longitudinally, galectin‐9 and CXCL10 outperformed the currently used laboratory marker, creatine kinase (CK), in distinguishing between juvenile DM patients with active disease and those in remission (area under the receiver operating characteristic curve [AUC] 0.86–0.90 for galectin‐9 and CXCL10; AUC 0.66–0.68 for CK). The sensitivity and specificity for active disease in juvenile DM was 0.84 and 0.92, respectively, for galectin‐9 and 0.87 and 1.00, respectively, for CXCL10. In 10 patients with juvenile DM who experienced a flare and were prospectively followed up, continuously elevated or rising biomarker levels suggested an imminent flare up to several months before the onset of symptoms, even in the absence of elevated CK levels. Galectin‐9 and CXCL10 distinguished between active disease and remission in adult patients with DM or NSM (*P* = 0.0126 for galectin‐9 and *P* < 0.0001 for CXCL10) and were suited for measurement in minimally invasive dried blood spots (healthy controls versus juvenile DM, *P* = 0.0040 for galectin‐9 and *P* < 0.0001 for CXCL10).

**Conclusion:**

In this study, galectin‐9 and CXCL10 were validated as sensitive and reliable biomarkers for disease activity in juvenile DM. Implementation of these biomarkers into clinical practice as tools to monitor disease activity and guide treatment might facilitate personalized treatment strategies.

## Introduction

Juvenile dermatomyositis (DM) is a rare, chronic systemic immune‐mediated disease with a high disease burden. In children with juvenile DM, the disease is characterized by inflammation of the skeletal muscles and skin, leading to muscle weakness and a pathognomonic skin rash. Vital organs such as the lung and heart can also be involved. Although the pathogenesis is still largely unknown, environmental and genetic factors may predispose children to the disease 1, 2, 3, 4, 5. The autoimmune process is characterized by a type I interferon signature and by infiltration of immune cells such as plasmacytoid dendritic cells, B cells, CD4+ T cells, and macrophages into the skin and muscle tissue 6, 7, 8, 9.

Children with juvenile DM are at risk of both under‐ and overtreatment due to a lack of reliable biomarkers that could be used to gauge the extent of disease activity. Current treatment guidelines recommend immunosuppression for at least 2 years, tapering steroids over the first year, and withdrawing treatment if a patient has been taken off steroids and has achieved disease remission with methotrexate (or an alternative disease‐modifying antirheumatic drug) for a minimum of 1 year 10, 11, 12. However, for some patients, this standardized regimen may not be optimal. Approximately 50% of patients do not respond to initial treatment or present with disease flares during follow‐up, resulting in additional tissue damage and impaired physical recovery 13, 14, 15. Of the other 50% of patients, some could likely benefit from a shorter treatment duration, taking into account that overtreatment with steroids can result in serious side effects in children, such as Cushing's syndrome, osteoporosis, and growth delay 16, 17, 18.

To determine the rate of medication tapering and to avoid both under‐ and overtreatment, objective measurement of disease activity and subclinical inflammation is crucial. However, validated and reliable biomarkers for disease activity in juvenile DM are lacking 19. Disease activity is currently assessed by a combination of muscle enzyme testing and clinical evaluation 10, 20, 21, 22; the latter depends on the experience of the health care professional and the patient's collaboration. Muscle enzymes, including serum creatine kinase (CK) activity, have been shown to correlate only moderately with disease activity in juvenile DM, and the erythrocyte sedimentation rate and C‐reactive protein level are rarely elevated in patients with juvenile DM 23, 24, 25. Lack of objective tools or biomarkers to monitor the response to therapy also hampers clinical trial design. Thus, there is an unmet need for an objective and reliable measure of disease activity.

Recently, in a cross‐sectional cohort of patients with juvenile DM, we demonstrated that 3 proteins, galectin‐9, CXCL10, and tumor necrosis factor receptor type II, can distinguish between juvenile DM patients with active disease and those in remission, with galectin‐9 and CXCL10 being the most discriminative markers 26, 27. CXCL10 and galectin‐9 can be produced by a variety of cells, both immune and nonimmune, upon stimulation with interferons 28, 29. CXCL10 has been recognized as a biomarker in several human autoimmune diseases, including myositis 29, 30, 31, 32, 33, whereas galectin‐9 has been investigated mainly as a biomarker in cancer and viral infections 28, 34. Reports on the role of galectin‐9 in autoimmunity are conflicting, suggesting either an attenuating or an aggravating effect on autoimmune manifestations in experimental models 35, 36. Its role in human autoimmune diseases has yet to be elucidated.

We aimed to validate galectin‐9 and CXCL10 as biomarkers for active disease in patients with juvenile DM, to examine their disease specificity in adult patients with DM, adult patients with nonspecific myositis (NSM), and patients with other systemic autoimmune diseases, to assess their potency in predicting flares, and to test the applicability of the biomarkers in minimally invasive dried blood spots, in order to aid broad implementation into clinical practice.

## Patients and methods

#### Cohorts

In total, 125 unique patients with juvenile DM from 3 independent cross‐sectional international cohorts and 1 Dutch prospective cohort participated in the present study, with inclusion between May 2001 and May 2017. Two large cohorts from Utrecht, The Netherlands and London, UK were used for validation of the biomarkers; a third smaller cohort from Singapore was used to assess international generalizability. An overview of all cohorts is shown in Table 1. The internal validation cohort (IVC) from Utrecht does not overlap with the previously reported discovery cohort 26. For specific questions, including disease specificity, longitudinal follow‐up, and measurements in dried blood spots, a combination of blood samples from the IVC and blood samples from new patients was used.

**Table 1:** Overview of the juvenile DM cohorts^a^

	Abbreviation	City, country	No. of patients	No. of samples	No. of active disease–remission paired samples
International validation cohorts					
External validation cohort	EVC	London, UK	61	79	16
Internal validation cohort	IVC; JDM NL	Utrecht, The Netherlands	47; 47	83; 58	26; 11
Asian cohort	JDM Sing	Singapore	12	13	–
Analysis‐specific subcohorts from Utrecht					
Systemic autoimmune disease cohort	–	Utrecht, The Netherlands	14	16	2
Longitudinal cohort	–	Utrecht, The Netherlands	28	286	–
Dried blood spot cohort	–	Utrecht, The Netherlands	7	10	–

a Data are listed as follows: for the London external validation cohort (EVC), see Figure 1, Supplementary Table 1, and Supplementary Figure 1; for the Utrecht internal validation cohort (IVC), see Figure 1, Supplementary Table 2, and Supplementary Figure 1; for the Juvenile Dermatomyositis The Netherlands (JDM NL) and Juvenile Dermatomyositis Singapore (JDM Sing) cohorts, see Figure 2 and Supplementary Table 4; for the systemic autoimmune disease cohort, see Figure 2 and Supplementary Table 5; for the longitudinal cohort, see Figure 3, Supplementary Table 6, and Supplementary Figure 2; for the dried blood spot cohort, see Figure 4 and Supplementary Table 7. All supplementary tables and figures are available on the *Arthritis & Rheumatology* web site at http://onlinelibrary.wiley.com/doi/10.1002/art.40881/abstract.

#### Participants

Patients with juvenile DM were included if they met the Bohan and Peter criteria for definite or probable juvenile DM 37, 38. The Childhood Myositis Assessment Scale (CMAS; scale 0–52) 39, Manual Muscle Testing of 8 muscle groups (MMT‐8; scale 0–80) 40, and physician's global assessment of disease activity (PhGA; scale 0–10) were recorded as clinical measures of muscle and global disease activity. In addition, cutaneous assessment tool (CAT) scores measuring the severity of skin disease (scale 0–116) 41 were recorded in Dutch and Singaporean patients. Disease remission was defined according to the updated criteria for clinically inactive disease and, in the case of missing data, was defined by clinical description 42. All other patients were considered to have active disease. Flares were defined as the combination of the following 3 items: a previous response to treatment with the decision to start tapering steroids, worsening of at least 1 of 3 clinical scores (CMAS, PhGA, and CAT) by ≥2 points, and the decision to start new immunosuppressive treatment or increase the current dose.

Adult patients with DM and those with NSM were classified according to the European Neuromuscular Centre criteria 43. Myositis was confirmed by biopsy unless typical skin manifestations of DM were present. Patients with cancer‐associated myositis were excluded. Disease activity was determined by combined evaluation of muscle strength with the Medical Research Council Muscle Scale 44, skin symptoms, and muscle enzyme levels. To determine the disease specificity of the biomarkers, different disease controls were added in the study, including pediatric and adult patients with systemic lupus erythematosus (SLE), pediatric patients with localized scleroderma, adult patients with eosinophilic fasciitis (EF), and pediatric and adult patients with hereditary proximal spinal muscular atrophy (SMA). All controls had either systemic inflammation, inflammation of the skin or muscles, or a noninflammatory neuromuscular disorder.

Patients with SLE fulfilled the American College of Rheumatology classification criteria for SLE 45. Active disease was defined as an SLE Disease Activity Index score of ≥4 of 105 46. Patients with localized scleroderma were diagnosed based on the typical clinical picture, with active disease being defined as a modified Localized Scleroderma Skin Severity Index (mLoSSi) score of ≥5 of 162 47. Patients with EF were diagnosed based on the clinical picture and histopathologic evaluation of skin biopsy specimens containing the fascia. As the mLoSSi may stay high in these patients due to the presence of extensive, irreversible sclerosis despite a reduction of inflammation, active disease was defined as a PhGA score of ≥5 (on 100‐mm visual analog scale) 47. Patients with hereditary proximal SMA, a progressive, noninflammatory neuromuscular disorder, were diagnosed by genetic confirmation of a homozygous loss of function of the survival motor neuron 1 gene 48; these patients served as disease controls. Adult healthy volunteers were included as healthy controls.

#### Ethics approval

The study was approved by the institutional ethics committees of the involved centers (UMC Utrecht [approval nos. METC 15‐191 and 12‐466], UK [approval no. MREC1/3/22], CHUV Lausanne, CHU Strasbourg, SingHealth centralized IRB, AMC Amsterdam) and conducted according to the Declaration of Helsinki. Written informed consent was obtained prior to inclusion in the study, both from patients and from parents or legal representatives when the patient was younger than 12 years old.

#### Blood samples

Blood was collected in serum tubes in accordance with the local study protocol (all participating centers). At the UMC Utrecht, blood samples were collected in sodium‐heparin tubes in addition to serum tubes. All samples were spun down and aliquoted within 4 hours after collection, and subsequently stored at −80°C until analyzed.

#### Measurement in dried blood spots

Dried blood spots were made by application of 50 μl sodium‐heparin full blood to each spot on Whatman 903 filter paper within 4 hours after the blood sample was obtained. Spotted filter papers were dried for 2 days at room temperature to mimic mail delivery times, and subsequently stored with desiccant in individual air‐tight polyethylene bags at −80°C under constant monitoring of humidity levels until analyzed. Two circles of 3.0 mm in diameter (containing ~3 μl of whole blood each) were punched from the central part of 1 spot and eluted in 100 μl buffer (phosphate buffered saline containing 5 ml/liter Tween 20, 10 gm/liter bovine serum albumin, and Complete protease inhibitor cocktail with EDTA [1 tablet per 25 ml buffer; Roche]) in 96‐well plates. Plates were sealed and placed overnight at 4°C on a microshaker (600 revolutions per minute) and were spun down at 2,100*g* for 2 minutes. The analysis was performed on the obtained eluate.

#### Biomarker analysis

Galectin‐9 and CXCL10 were measured in 50 μl of serum, plasma, or eluate by multiplex assay (xMAP; Luminex). CXCL10 was measured in undiluted material. Galectin‐9 was measured in 10× diluted plasma or serum, except in the serum/plasma samples paired with dried blood spots (in which case galectin‐9 was measured undiluted from the eluate and serum/plasma). The multiplex immunoassay was performed as described previously 49. Heterophilic immunoglobulins were preabsorbed from all samples with HeteroBlock (Omega Biologicals). Acquisition was performed with a Bio‐Rad FlexMAP3D in combination with xPONENT software version 4.2 (Luminex). Data analysis was performed with Bioplex Manager version 6.1.1 (Bio‐Rad).

Between measurement of the internal and external validation cohorts in 2015, the recombinant protein for galectin‐9 was replaced, which affected the standard curve. Therefore, absolute values between these cohorts may not be comparable. Since 2015, the interassay variability has been negligible 50. All biomarker analyses were performed at the UMC Utrecht, thereby minimizing intercenter variation. Treating physicians were blinded with regard to biomarker levels, and technicians performing the multiplex assay were blinded with regard to clinical data.

#### Statistical analysis

Basic descriptive statistics were used to describe the patient population. Statistical analyses were performed using either GraphPad Prism version 7.0 or SPSS Statistics version 21 (IBM). Correlations were assessed using Spearman's rank correlation coefficients. For comparisons between 2 groups, the Mann‐Whitney U test (unpaired analysis) or Wilcoxon's matched‐pairs signed rank test (paired analysis) was used. For comparisons between multiple groups, nonparametric variants of analysis of variance (ANOVA) with post hoc correction for multiple testing were used (Dunn's post hoc test for Kruskal‐Wallis, and Šídák's or Tukey's post hoc test for 2‐way ANOVA, as appropriate). Multiplicity‐adjusted *P* values less than 0.05 were considered significant.

To assess diagnostic accuracy, area under the receiver operating characteristic (ROC) curves (AUCs) were constructed. Cutoff values for the diagnostic accuracy of galectin‐9 and CXCL10 were determined based on the maximal Youden's Index, with a sensitivity of at least 80%.

## Results

#### Cross‐sectional validation of galectin‐9 and CXCL10

To validate the biomarker potential of galectin‐9 and CXCL10, we measured the proteins in blood samples from patients with juvenile DM from 2 independent validation cohorts: an external validation cohort (EVC) from London and an internal validation cohort (IVC) from Utrecht. The clinical characteristics of these cohorts are shown in Supplementary Tables 1 and 2 (available on the *Arthritis & Rheumatology* web site at http://onlinelibrary.wiley.com/doi/10.1002/art.40881/abstract). As observed in the previously reported discovery cohort 26, the levels of galectin‐9 and CXCL10 were significantly higher in patients with active disease compared to patients in remission (*P* < 0.0001) (results in Supplementary Figures 1A and B [http://onlinelibrary.wiley.com/doi/10.1002/art.40881/abstract]). The levels were highest at the time of diagnosis (before treatment), decreased steadily under treatment, and were comparably low in remission regardless of whether the patient was receiving or not receiving medication while in remission (Figures 1A and B).

**Figure 1:**
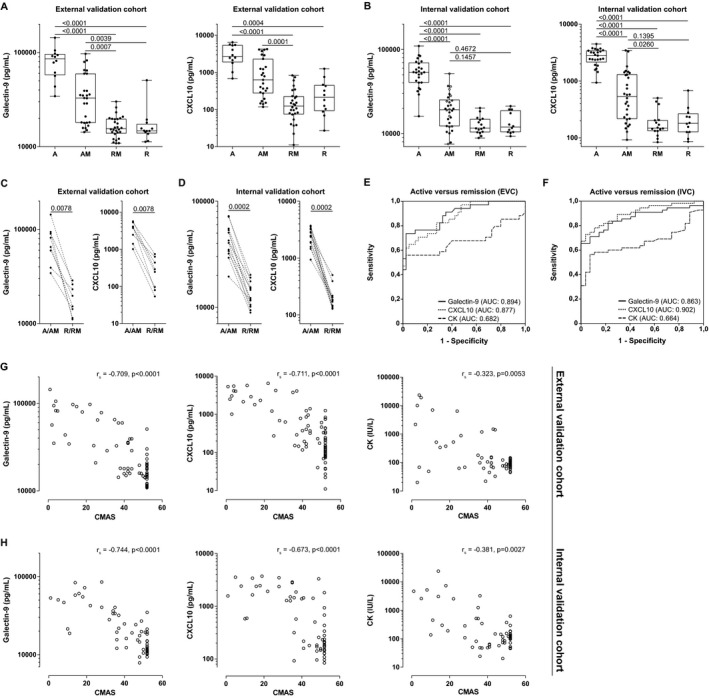
Cross‐sectional validation of galectin‐9 and CXCL10 as biomarkers for disease activity in juvenile dermatomyositis (DM) in 2 independent validation cohorts. **A** and **B,** Measurement of galectin‐9 and CXCL10 by multiplex immunoassay in serum from patients with active disease before start of treatment (group A), active disease while receiving medication (group AM), in remission while receiving medication (group RM), and in remission while not receiving medication (group R), in the external validation cohort (EVC) (n = 12 group A, n = 27 group AM, n = 28 group RM, n = 12 group R) (**A**) and the internal validation cohort (IVC) (n = 25 group A, n = 30 group AM, n = 16 group RM, n = 12 group R) (**B**). In group AM, 3 samples from 1 patient (from different time points at least 3 months apart) and 2 samples from 6 patients (from different time points 2–11 months apart) were included. Data are shown as box plots. Each box represents the interquartile range. Lines inside the boxes represent the median (log scale). Lines outside the boxes represent the 10th and 90th percentiles. Symbols represent individual patients. Multiplicity‐adjusted *P* values were determined by Kruskal‐Wallis test with Dunnett's post hoc test. **C** and **D**, Measurement of galectin‐9 and CXCL10 in paired samples from individual patients (regardless of treatment status) during active disease and remission, from the EVC (median time between samples 23 months) (**C**) and IVC (median time between samples 12 months) (**D**). *P* values were determined by Wilcoxon's matched‐pairs signed rank test. **E** and **F,** Area under the receiver operating characteristic (ROC) curves (AUCs) for diagnostic accuracy of galectin‐9, CXCL10, and creatine kinase (CK) in patients (regardless of treatment status) from the EVC (**E**) and IVC (**F**). Only patients with complete data for the specific ROC curve were included. **G** and **H,** Spearman's rank correlations of galectin‐9, CXCL10, and CK levels with Childhood Myositis Assessment Scale (CMAS) scores in the EVC (n = 79) (**G**) and IVC (n = 61) **(H)**.

The wide range of biomarker levels in the group of juvenile DM patients with active disease who were on treatment corresponded to a wide range of clinical disease activity scores within this group (CMAS scores ranging 3–44 in the EVC and 10–52 in the IVC; PhGA scores ranging 2–8 in the EVC and 1–9 in the IVC). Based on the levels of both galectin‐9 and CXCL10, we were able to differentiate patients with active disease while receiving medication from patients in remission while receiving medication (Figures 1A and B), which is clinically important to assess the response to treatment. Paired analysis within individual patients, in which we compared samples from a period of active disease and from a period of remission in each patient, showed decreasing biomarker levels in response to therapy and confirmed the high discriminative power of both proteins (each *P* = 0.0078 in the EVC and *P* = 0.0002 in the IVC) (Figures 1C and D).

To further assess the discriminative power of galectin‐9 and CXCL10 for distinguishing between a status of active disease and a status of remission in juvenile DM, we examined the AUCs in the 2 separate cohorts. In comparing active disease and remission in patients regardless of their treatment status, the levels of galectin‐9 and CXCL10 had AUCs of 0.894 and 0.863, respectively, in the EVC and 0.877 and 0.902, respectively, in the IVC (Figures 1E and F, and Supplementary Table 3 [online at http://onlinelibrary.wiley.com/doi/10.1002/art.40881/abstract]).

To take into account the effect of treatment, we also assessed the AUC for differentiating active disease from disease remission in patients who were taking medication. During treatment, the levels of galectin‐9 and CXCL10 had AUCs of 0.844 and 0.776, respectively, in the EVC and 0.860 and 0.840, respectively, in the IVC (see xSupplementary Figures 1C and D and Supplementary Table 3 [http://onlinelibrary.wiley.com/doi/10.1002/art.40881/abstract]). Moreover, galectin‐9 and CXCL10 performed better than the current standard laboratory marker, CK, in both cohorts (AUCs for CK, 0.682 in the EVC and 0.662 in the IVC).

To calculate the optimal cutoff value for distinguishing active disease from disease remission, we analyzed the ROC curves in the IVC, as blood samples from this cohort were assessed according to the most recently optimized and standardized protocol of the multiplex immunoassay 50. Based on the coordinates of this ROC curve, we determined the cutoff values for discriminating active disease from remission, yielding a cutoff value of 19,396 pg/ml for galectin‐9 and 805 pg/ml for CXCL10, with a high sensitivity (0.84 for galectin‐9 and 0.87 for CXCL10) and a high negative predictive value (0.83 for galectin‐9 and 0.87 for CXCL10) (Table 2); these values ensured a low risk of ongoing inflammation in the case of a test result that was below the cutoff. The specificity of the galectin‐9 and CXCL10 cutoff levels was 0.92 and 1.00, respectively, and the positive predictive value was 0.93 and 1.00, respectively.

**Table 2:** Sensitivity, specificity, NPV, and PPV of the determined cutoff values for diagnostic accuracy of galectin‐9 and CXCL10 in the juvenile dermatomyositis internal validation cohort^a^

	Galectin‐9	CXCL10
Cutoff value, pg/ml	19,396	805
Sensitivity	0.839	0.871
Specificity	0.923	1.000
NPV	0.828	0.867
PPV	0.929	1.000

a Cutoff values for galectin‐9 and CXCL10 were determined based on the maximal Youden's Index with a sensitivity of >0.80, in order to ensure a low risk of ongoing active inflammation with a biomarker value below the set cutoff. Only 1 sample per patient per category (active disease or in remission) was included in the analysis (i.e., the cohort designated “JDM NL” [Juvenile Dermatomyositis The Netherlands], as shown in Figure 2 and Supplementary Table 5 on the *Arthritis & Rheumatology* web site at http://onlinelibrary.wiley.com/doi/10.1002/art.40881/abstract]). NPV = negative predictive value; PPV = positive predictive value.

Consistent with the previously reported discovery cohort 26, the levels of galectin‐9 and CXCL10 correlated strongly with 3 clinical scores of global or muscle disease activity: the PhGA, the CMAS, and the MMT‐8. The correlation coefficients for association with either of the biomarkers, which ranged between 0.67 and 0.81 (*P* < 0.0001), were notably higher than those for CK (r_s_ = 0.32–0.51, *P* < 0.01) (Figures 1G and H, and Supplementary Figures 1E–G [http://onlinelibrary.wiley.com/doi/10.1002/art.40881/abstract]). Thus, these results in 2 independent validation cohorts validate galectin‐9 and CXCL10 as strong biomarkers for disease activity in patients with juvenile DM, outperforming the currently used laboratory marker CK.

To assess the international generalizability of galectin‐9 and CXCL10, we tested the biomarkers in a small cohort of patients with juvenile DM from a different geographic region (i.e., Singapore). Observations in this cohort confirmed the discriminative potential of galectin‐9 and CXCL10 between active disease and remission, and their levels were comparable to those seen in the IVC (*P* = 0.0006 for galectin‐9 and *P* = 0.0025 for CXCL10) (Figures 2A and B).

**Figure 2:**
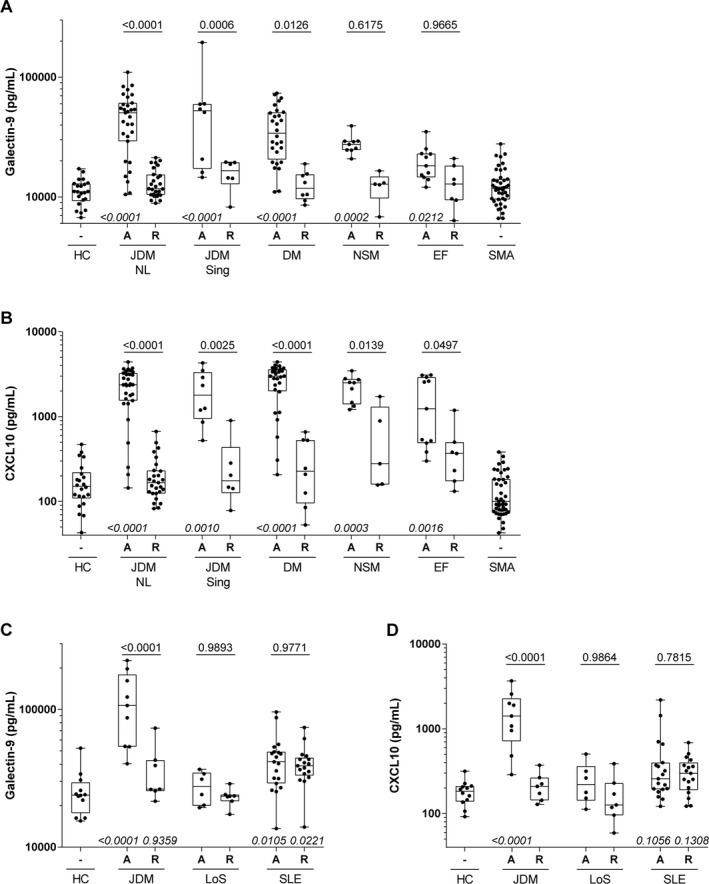
Biomarker potential of galectin‐9 and CXCL10 in adult inflammatory myopathies and systemic autoimmune diseases with skin involvement. **A** and **B,** Galectin‐9 (**A**) and CXCL10 (**B**) were measured in serum samples from patients with juvenile dermatomyositis from The Netherlands (JDM NL) (the internal validation cohort [IVC]) and Singapore validation cohort (JDM Sing), adult patients with DM, adult patients with nonspecific myositis (NSM), adult patients with eosinophilic fasciitis (EF), a mixed cohort of adult and juvenile patients with hereditary proximal spinal muscular atrophy (SMA), and adult healthy controls (HC). **C** and **D,** Galectin‐9 (**C**) and CXCL10 (**D**) were measured in serum samples from the Dutch juvenile DM cohort, juvenile patients with localized scleroderma (LoS), a mixed cohort of juvenile and adult patients with systemic lupus erythematosus (SLE), and adult healthy controls. In **A–D,** patients were stratified into 2 groups based on disease activity (active [A] or in remission [R] regardless of treatment status). Only 1 sample per patient per activity group was included in the analysis; therefore, the numbers of patients in the IVC differ from those in Figure 1. Data are shown as box plots. Each box represents the interquartile range. Lines inside the boxes represent the median (log scale). Lines outside the boxes represent the 10th and 90th percentiles. Symbols represent individual patients. Multiplicity‐adjusted *P* values above boxes are for comparison between active disease and remission, by 2‐way analysis of variance with Šídák's post hoc test for multiple comparisons. Multiplicity‐adjusted *P* values below boxes are for comparison between each disease group and healthy controls, by Kruskal‐Wallis test with Dunnett's post hoc test for multiple comparisons. *P* values >0.999 are not shown.

#### Disease specificity of galectin‐9 and CXCL10

We next investigated the disease specificity of galectin‐9 and CXCL10 and explored the applicability of each as a biomarker in adult patients with DM or NSM and patients with other systemic autoimmune diseases. The biomarkers were first measured in a cohort of adult patients with DM (n = 36), patients with NSM (n = 14), and patients with EF (n = 18), as well as 43 disease control patients with SMA, a genetic neuromuscular disorder without systemic inflammation, and 22 healthy controls (the characteristics of these subjects are listed in Supplementary Table 4 [http://onlinelibrary.wiley.com/doi/10.1002/art.40881/abstract]). The levels of both galectin‐9 and CXCL10 were elevated in adult patients with active DM (*P* < 0.0001), patients with NSM (*P* < 0.0003), and patients with EF (*P* < 0.05) as compared to healthy controls. Both biomarkers distinguished between active disease and remission in the adult DM cohort (*P* = 0.0126 and *P* < 0.0001 for galectin‐9 and CXCL10, respectively), and CXCL10 was also discriminative for disease activity in patients with NSM (*P* = 0.0139) and those with EF (*P* = 0.0497) (Figures 2A and B). As expected, the biomarkers were not elevated in control patients with SMA.

A second cohort consisted of pediatric and adult patients with 2 other systemic immune‐mediated diseases: localized scleroderma (n = 15) and SLE (n = 36) (the characteristics of these patients are listed in Supplementary Table 5 [http://onlinelibrary.wiley.com/doi/10.1002/art.40881/abstract]). In patients with localized scleroderma and those with SLE, the 2 biomarkers did not distinguish significantly between active disease and remission, but galectin‐9 levels in patients with SLE were elevated compared to healthy controls (*P* = 0.0105) (Figures 2C and D).

Thus, these results demonstrate that galectin‐9 and CXCL10 are applicable as biomarkers for disease activity in both pediatric and adult patients with myositis.

#### Prospective analysis and flare prediction

**Figure 3:**
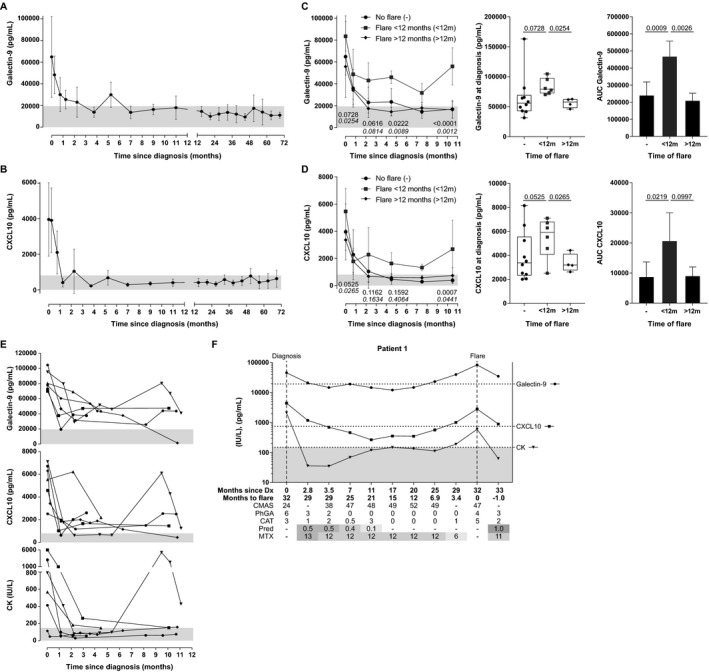
Galectin‐9 and CXCL10 serum levels from longitudinal follow‐up of patients with juvenile dermatomyositis (DM) in a prospective cohort. **A** and **B**, Dynamics of galectin‐9 (**A**) and CXCL10 (**B**) serum levels up to 6 years after juvenile DM diagnosis in 15 patients without flares. The first sample was obtained a maximum of 6 months after treatment start. Both patients with and those without intensification of therapy within the first 3 months were included. Each point contains between 3 and 13 samples, pooled over the time span around the data point. The median interval between 2 samples from a patient was 3.6 months. Per patient, 4–14 samples (median 9) were included. Values are the mean ± SD (linear scale). **C** and **D**, Galectin‐9 (**C**) and CXCL10 (**D**) serum levels in longitudinal samples from juvenile DM patients with a flare within the first year (<12m) (n = 6), after the first year (>12m) (n = 7), or without flares (n = 15) (same patients as in **A** and **B**). Only patients with a first sample obtained a maximum of 6 months after treatment start were included. Left, Longitudinal data (mean ± SD) within the first year. Multiplicity‐adjusted *P* values, by 2‐way analysis of variance (ANOVA) with Tukey's post hoc test, were for flare <12 months versus no flare (top) or flare <12 months versus flare >12 months (bottom) Middle, Galectin‐9 and CXCL10 levels at diagnosis, before treatment start. Data are shown as box plots. Boxes represent the interquartile range, lines inside the boxes show the median, and lines outside the boxes show the 10th and 90th percentiles. Symbols represent individual patients. All *P* values, by 2‐way ANOVA with Tukey's post hoc test, were corrected. Right, Total area under the receiver operating characteristic curves (AUCs) for each group, calculated by the trapezoidal method. Values are the mean and 95% confidence interval. *P* values were determined by one‐way ANOVA with Tukey's post hoc test. **E**, Galectin‐9, CXCL10, and creatine kinase (CK) serum levels in 6 individuals with a flare within the first year after treatment start. In **A–F,** Gray shading indicates the previously determined cutoffs for galectin‐9 and CXCL10 (19,396 pg/ml and 805 pg/ml, respectively) and the standard cutoff for CK (150 IU/liter). **F**, Levels of galectin‐9, CXCL10, and CK measured longitudinally in an individual with a disease flare after the first year. Broken horizontal lines indicate the previously determined cutoffs for galectin‐9, CXCL10, and CK. Biomarker levels are shown on a log scale. Shading in the rows for prednisone (Pred) and methotrexate (MTX) represent the relative medication dose (in mg/kg/day for prednisone; in mg/m^2^/week for MTX). Dark gray shading = high dose; lighter gray shading = low dose. Dx = diagnosis; CMAS = Childhood Myositis Assessment Scale; PhGA = physician's global assessment; CAT = cutaneous assessment tool.

To determine the prognostic value of galectin‐9 and CXCL10 during clinical follow‐up in patients with juvenile DM, we measured the biomarkers in a prospective cohort of 28 patients, with a median follow‐up time of 2.8 years per patient (the characteristics of these patients are listed in Supplementary Table 6 [http://onlinelibrary.wiley.com/doi/10.1002/art.40881/abstract]). First, we established the biomarker dynamics after diagnosis in 15 patients who reached sustained remission within the first months of treatment and did not have a flare later. The biomarker levels quickly declined after the start of treatment, reached levels below the previously determined cutoff value within several months, and remained low in remission (the “No flare” group, shown in Figures 3A and B). The biomarker dynamics in patients with a flare after the first year (the “Flare >12 months” group; n = 7) were similar to those in patients without flares (Figures 3C and D). However, patients who experienced a disease flare in the first year after the start of treatment (the “Flare <12 months” group; n = 6) had significantly higher biomarker levels at diagnosis than did patients with later flares (*P* = 0.0254 for galectin‐9 and *P* = 0.0265 for CXCL10) (Figures 3C and D). In addition, these patients who experienced a flare at <12 months had elevated biomarker levels over the entire first year (Figures 3C–E). In contrast to the 2 biomarkers, CK activity normalized in 5 of 6 patients (Figure 3E).

To assess the predictive value of the biomarkers for flares after the first year, we analyzed 4 patients for whom longitudinal samples were available within 7 months before a flare (Figure 3F and Supplementary Figure 2 [http://onlinelibrary.wiley.com/doi/10.1002/art.40881/abstract]). In patients 1 and 2, raised levels of galectin‐9 and CXCL10 (even while remaining below the cutoff level) were observed from up to 7 months prior to the flare, with levels that were above the cutoff value up to 6 months prior to the flare for galectin‐9 and up to 3 months prior to the flare for CXCL10. These biomarker fluctuations were observed even before clinical symptoms of a flare became apparent. In patients 3 and 4, persistently borderline cutoff values were observed for galectin‐9 and CXCL10 in the 12 months prior to occurrence of a flare, and biomarkers were elevated above the cutoff during the flare. In contrast, CK levels did not increase prior to or during a flare in patient 4, and did not demonstrate an increase until the occurrence of a flare in patients 2 and 3. Only in patient 1 did the CK level steadily increase by 3 months prior to a flare. It was also observed that galectin‐9 and CXCL10 levels stayed high during continued disease activity after the start of the flare in patients receiving medication, while in 3 of 4 individuals, the CK level decreased to within normal limits by the first time point following the start of the clinical flare, despite continued disease activity.

Thus, these results suggest that persistently high or rising galectin‐9 and CXCL10 levels above their cutoff values may be indicative of ongoing (sub)clinical inflammation or an imminent flare, even with a lack of clinical symptoms or elevated CK levels.

#### Levels of galectin‐9 and CXCL10 in dried blood spots

**Figure 4:**
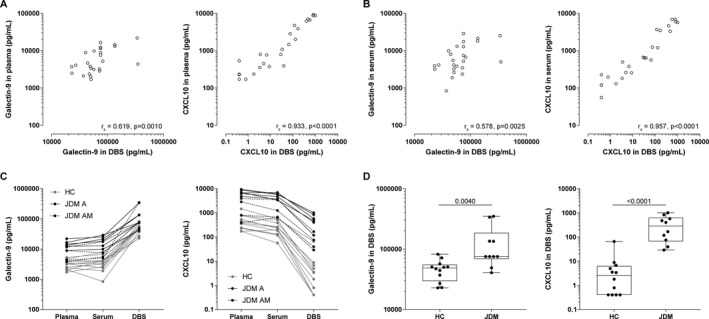
Measurement of galectin‐9 and CXCL10 levels in dried blood spots (DBS) as compared to paired plasma and serum samples from patients with active juvenile dermatomyositis (DM). **A** and **B,** Correlations between biomarker levels in the plasma (**A**) and serum (**B**) and in DBS (on a double log scale) were assessed using Spearman's correlation coefficients. **C**, Paired representation of the biomarker levels in the plasma, serum, and DBS from healthy controls (HC), patients with active juvenile DM pretreatment (JDM A), and patients with active juvenile DM while receiving medication (JDM AM) are shown. **D**, Biomarker levels in DBS were compared between healthy controls and patients with active juvenile DM. *P* values were determined by Mann‐Whitney U test.

To facilitate minimally invasive (at‐home) biomarker assessment and broad clinical applicability with centralization of diagnostic cores, we assessed galectin‐9 and CXCL10 measurements in dried blood spots and paired plasma and serum samples (the patients’ characteristics are shown in Supplementary Table 7 [http://onlinelibrary.wiley.com/doi/10.1002/art.40881/abstract]). Correlation between the biomarker levels in the circulation and biomarker levels in dried blood spots was higher for CXCL10 (r_s_ = 0.93 in plasma and r_s_ = 0.96 in serum) than for galectin‐9 (r_s_ = 0.62 in plasma and r_s_ = 0.58 in serum) (Figures 4A and B). Galectin‐9 and CXCL10 levels were similar in the plasma and serum (Figure 4C). Both galectin‐9 and CXCL10, as measured in dried blood spots, were capable of discriminating between patients with active juvenile DM and healthy controls (*P* = 0.0040 and *P* < 0.0001, respectively) (Figure 4D), with the healthy control subjects having biomarker levels that were similar to those in patients with juvenile DM in remission (Figure 2). Thus, measurements of both galectin‐9 and CXCL10 in dried blood spots are suitable as biomarkers for juvenile DM disease activity.

## Discussion

In this study, galectin‐9 and CXCL10 were validated as strong, reliable, and sensitive biomarkers for disease activity in juvenile DM, and both were identified as promising biomarkers both in adult patients with DM and in adult patients with NSM. The levels of galectin‐9 and CXCL10 strongly distinguished between juvenile DM patients with active disease and juvenile DM patients in remission, even when the patient was receiving immunosuppressive treatment. Furthermore, we showed that galectin‐9 and CXCL10 were relatively specific for autoinflammatory myopathies in adult and pediatric patients, as their levels were not as highly increased or did not differentiate between active disease and remission in other autoimmune diseases such as localized scleroderma and SLE. Both cross‐sectionally and longitudinally, galectin‐9 and CXCL10 outperformed CK, which is commonly used as a laboratory marker for disease activity and is one of the current criteria for determining clinically inactive disease in juvenile DM 42, 51. Continuously elevated or rising biomarker levels, as determined in a prospective patient cohort, may be indicative of an imminent disease flare up to several months before clinical symptoms, even in the absence of elevated CK levels. The biomarkers may therefore be promising to use in longitudinal follow‐up of patients for monitoring of disease activity.

Furthermore, our results showed that galectin‐9 and CXCL10 can be reliably measured in the plasma, serum, and minimally invasive dried blood spots from patients with juvenile DM. It has recently been shown that capillary concentrations of CXCL10 correlate with venous concentrations; for galectin‐9, this has not yet been established 52. The moderate correlation between circulating levels of galectin‐9 and levels of galectin‐9 in dried blood spots could be attributed to either liberation of intracellularly stored galectin‐9 and/or release from its carrier proteins upon elution and dilution.

This study has several strengths. Although many biomarkers are being identified for a variety of diseases, only a few have been implemented into clinical practice, due to a lack of reproducibility and diagnostic accuracy. However, the levels of galectin‐9 and CXCL10 have a high discriminative power and strong, reproducible correlation with disease activity. Thanks to a large international collaborative effort, and despite the rarity of the disease, we have been able to extensively validate galectin‐9 and CXCL10 as biomarkers in a large number of patients with juvenile DM from 3 independent cross‐sectional cohorts. The additional analyses in a prospective cohort of patients with juvenile DM with a long follow‐up time added important information on the value of galectin‐9 and CXCL10 in clinical follow‐up. In addition to the clinical validation in this study, the biomarkers have undergone a technical validation at the diagnostic department of the UMC Utrecht, which has demonstrated the stability of the biomarkers and reproducibility of the measurements. In addition, we have explored a minimally invasive diagnostic method of measuring the biomarkers in dried blood spots.

The findings of this study need to be interpreted carefully, taking into account the observational nature of the data and the use of a combination of clinical scores and CK levels (the Paediatric Rheumatology International Trials Organisation criteria for clinically inactive disease in juvenile DM) as the gold standard for assessment of disease activity in juvenile DM 42, 51. Importantly, measurement of galectin‐9 and CXCL10 levels can complement, but not replace, clinical assessment by experienced health care professionals. However, both biomarkers outperformed the currently used marker, CK, a finding that underscores the gains that can be achieved by introducing the new biomarkers into clinical practice.

A recent study using the SOMAscan assay also identified both galectin‐9 and CXCL10 among the top up‐regulated proteins in juvenile DM, correlating with disease activity as assessed by the PhGA 53. CXCL10 levels were previously shown to correlate with disease activity in juvenile DM 26, 30, 31, 32, 54, and CXCL10 is well known to be an interferon‐inducible chemokine that can be elevated in other types of myositis and autoimmune diseases 29, 33. In our study, galectin‐9 was a specific biomarker for inflammatory myopathies. In patients with juvenile DM, high circulating interferon‐α levels have been found, and in one group of patients with juvenile DM, more than 75% of patients had a positive interferon signature 55, 56. Circulating galectin‐9 and CXCL10 levels could therefore be a direct reflection of active, interferon‐driven inflammation, which is supported by a recent study in which galectin‐9 was demonstrated to be a marker for the interferon signature in SLE and antiphospholipid syndrome 57.

Since the levels of these biomarkers are known to correlate with the extent of disease activity in various types of tissue, local tissue cells are the main candidate producers of the proteins. Indeed, galectin‐9 can be detected not only in the circulation, but also locally within inflamed muscle and skin, where it is mainly present in activated tissue macrophages and capillary endothelial cells (data not shown). A similar expression pattern, in tissue mononuclear cells and endothelial cells, was previously demonstrated for CXCL10 58, 59. Local biomarker production within the inflamed tissue is consistent with our previous observation that the biomarker levels slowly decline after stem cell transplantation, as tissue‐infiltrating immune cells (and endothelial cells) are likely to be less affected by immune‐ablative preconditioning than are circulating immune cells 27.

Implementation of galectin‐9 and CXCL10 into clinical practice, as tools to monitor disease activity and guide treatment, might enable personalized treatment strategies for patients with juvenile DM. It is an advantage that both biomarkers performed equally well in our study, suggesting that diagnostic centers can decide to use their biomarker of choice depending on its availability and feasibility. Biomarker levels below the set cutoff value reflect the absence of disease activity, which could allow tapering of immunosuppressive medication. Rising or persistently high levels might be indicative of an insufficient response to therapy and/or an imminent flare, even in the absence of clinical symptoms or elevated CK levels, possibly reflecting subclinical inflammation. Elevated biomarker levels might therefore indicate the need for intensification of treatment or slower tapering of steroids. With this envisioned personalized treatment strategy, we could respond to important patient‐reported needs: a recently conducted patient survey by Cure JM, a US patient organization for juvenile myositis, has shown that “predictors for disease flares” and “new treatments, less side effects” are 2 of the top 3 research priorities chosen by patients 60.

Galectin‐9 and CXCL10 may also provide an objective outcome measure for response to therapy in future clinical trials that would be assessing novel therapeutics. Our study has shown that galectin‐9 and CXCL10 levels in dried blood spots correlate with venous levels and could differentiate patients with active juvenile DM from healthy controls. Longitudinal assessment of these biomarkers via dried blood spots, which requires further study, has potential for high utility in the future, since dried blood spots can be sampled at home by simple capillary finger‐prick. Since protein levels in dried blood spots remain remarkably stable over time, even at room temperature 61, 62, samples of dried blood spots can be sent to a diagnostic center through regular mail. This enables at‐home diagnostics and centralization of diagnostic cores for both clinical care and multicenter studies. It also ensures maximum accessibility of the biomarker measurements for non–expert medical centers, which can also facilitate care in rural areas.

Galectin‐9 and CXCL10 measurements could add important information to the complex differential diagnosis of muscle symptoms during follow‐up, and might aid in discriminating between steroid‐induced myopathy, noninflammatory muscle pain, and muscle inflammation, all of which require different treatment strategies. In these complicated cases, in particular, the biomarkers may also help abrogate the need for invasive diagnostic muscle biopsy or costly magnetic resonance imaging scans, which can sometimes require sedation in young children. This specific potential use of these biomarkers will have to be further investigated in additional prospective studies. In addition, future prospective studies will have to point out 1) whether one biomarker may be superior to the other in answering specific clinical questions concerning juvenile DM, 2) whether the biomarkers are able to detect mild disease activity, 3) whether the biomarkers also have prognostic value in adult patients with myositis, and 4) whether biomarker‐guided disease management will improve the outcomes in patients with juvenile DM.

In conclusion, galectin‐9 and CXCL10 were identified and extensively validated as strong, reliable, and sensitive biomarkers for disease activity in juvenile DM. Measurement of these biomarkers might facilitate personalized treatment strategies for patients with juvenile DM, by providing a diagnostic monitoring tool to guide treatment.

## Author Contributions

All authors were involved in drafting the article or revising it critically for important intellectual content, and all authors approved the final version to be published. Drs. van Royen‐Kerkhof and van Wijk had full access to all of the data in the study and take responsibility for the integrity of the data and the accuracy of the data analysis.

### Study conception and design

Wienke, Bellutti Enders, Lim, Otten, Fritsch‐Stork, de Jager, de Visser, Arkachaisri, van der Kooi, Nierkens, Wedderburn, van Royen‐Kerkhof, van Wijk.

### Acquisition of data

Wienke, Bellutti Enders, Lim, Mertens, van den Hoogen, Wijngaarde, Yeo, Meyer, Otten, Fritsch‐Stork, Kamphuis, Hoppenreijs, Armbrust, van den Berg, Hissink Muller, Tekstra, Hoogendijk, Deakin, van Roon, van der Pol, Nistala, Pilkington, Arkachaisri, Radstake, van der Kooi, Nierkens, Wedderburn, van Royen‐Kerkhof, van Wijk.

### Analysis and interpretation of data

Wienke, Bellutti Enders, Lim, Mertens, de Jager, de Visser, van der Kooi, Nierkens, Wedderburn, van Royen‐Kerkhof, van Wijk.
