# Supplementary material for: Galectin‐9 and CXCL10 as Biomarkers for Disease Activity in Juvenile Dermatomyositis: A Longitudinal Cohort Study and Multicohort Validation
Source: Arthritis Rheumatol. 2019 Mar 12;71(8):1377–90. doi: 10.1002/art.40881 (PMC6973145; doi:10.1002/art.40881)
Supplement: Supplementary file 1 [file ART-71-1377-s001.docx]

**SUPPLEMENTARY MATERIAL**

1. Supplementary figure and table legends

2. Supplementary figure 1

3. Supplementary figure 2

4. Supplementary table 1

5. Supplementary table 2

6. Supplementary table 3

7. Supplementary table 4

8. Supplementary table 5

9. Supplementary table 6

10. Supplementary table 7

**SUPPLEMENTARY FIGURE AND TABLE LEGENDS**

**Supplementary Figure 1. Cross-sectional validation of galectin-9 and CXCL10 as biomarkers for disease activity in juvenile DM (JDM) in two independent validation cohorts.**

(**A&B**) Galectin-9 and CXCL10 levels were measured in serum samples from juvenile DM patients by multiplex immunoassay. Patients were stratified into two groups based on disease activity, regardless of medication. Medians and interquartile ranges are shown, on a log scale. Mann-Whitney U test. (**A)** External validation cohort (EVC) (A/AM: n=39, R/RM: n=40). (**B**) Internal validation cohort (IVC) (A/AM: n=55, R/RM: n=28). (**C&D**) ROC curves of galectin-9, CXCL10, and CK in the EVC (**C**) and IVC (**D**). Only patients with a complete dataset for the specific ROC curve were included in the analysis. ROC curves for patients on medication. Statistic details of the ROC curve analysis are shown in supplementary table 3. (**E&F) C**orrelation of galectin-9, CXCL10 and CK with the PGA (**E**) and MMT-8 (**F**) in the EVC. Spearman rank correlation, n=79. (**G**) Correlation of galectin-9, CXCL10 and CK with the PGA in the IVC. Spearman rank correlation, n=67*. A = active pre-treatment; AM = active on medication; RM = remission on medication; R = remission off medication; rs= Spearman r; PGA = physician’s global assessment; MMT-8 = manual muscle testing scores of 8 muscle groups. Patient characteristics are shown in table 1 and 2.*

**Supplementary Figure 2. Galectin-9, CXCL10, CK in individual patients with disease flares after the first year.**

Galectin-9 and CXCL10 were measured in longitudinal samples from 3 patients with juvenile DM (JDM) with a disease flare after the first year. Each panel represents one patient. Dotted lines indicate the previously determined cutoff values for galectin-9 and CXCL10 (19396 pg/mL and 805 pg/mL, respectively). Gray shading indicates the cutoff for CK (150 IU/L). Time is in months. *CK = creatine kinase, IU = international units, Dx = diagnosis,CMAS = childhood myositis assessment scale, PGA = Physician’s global assessment, CAT = cutaneous assessment tool, Pred = prednisone (mg/kg/day), MP = methylprednisolone (Pulse= 3x 20 mg/kg/day), MTX = methotrexate (mg/m2/week), IVIG = intravenous immunologlobulins (Pulse= 2 g/kg per month), Tacro =Tacrolimus, MMF = mycophenolate mofetil, Cyclophos = Cyclophosphamide (Pulse= 750 mg/m2 per month).Patient characteristics are shown in supplementary table 7.*

**Supplementary Table 1. Patient characteristics of cross-sectional external validation cohort from London.**

Patients were stratified into four groups based on disease activity and use of medication: active pre-treatment (“A”), active on medication (“AM”), remission on medication (“RM”), remission off medication (“R”). Unless indicated otherwise, values represent median (range). The CMAS and MMT-8 were used to assess muscle strength. ANA were detected by indirect immunofluorescence on Hep-2 cells. All patients in remission had all clinical data available (PGA, CK, CMAS and MMT8). The missing data in the A/AM groups did not affect their classification due to redundancy of de data (CMAS and MMT8). 1 patient in the “A” group had started treatment less than 1 week before sampling and was therefore still included in the pre-treatment group. In the “R” group the median time between stopping immunosuppressive medication and sampling was 12.4 months, with a range of 0.9-93.9 months. *NR=not recorded, CMAS =childhood myositis assessment scale, PGA = physician’s global assessment, MMT-8 = manual muscle testing of 8 muscle groups, ANA = anti-nuclear antibodies, MSA = myositis-specific antibodies, IV = intravenous, CK =creatine kinase, AST = aspartate aminotransferase, ALT = alanine aminotransferase, LDH = lactate dehydrogenase, CRP = C-reactive protein, ESR = erythrocyte sedimentation rate, IU= international units*

**Supplementary Table 2. Patient characteristics of cross-sectional internal validation cohort.**

Patients were stratified into four groups based on disease activity and use of medication: active pre-treatment (“A”), active on medication (“AM”), remission on medication (“RM”), remission off medication (“R”). Unless indicated otherwise, values represent: median (range). The CMAS was used to assess muscle strength. ANA were detected by indirect immunofluorescence on Hep-2 cells. 8 out of 55 active patients had a CMAS>47, but were nonetheless defined as active based on active skin disease and/or active arthritis, rendering an elevated PGA. 3 of these patients also had an elevated CK despite a normal CMAS. All patients in remission were sure to be in remission even with missing data: 1 patient did not have a CMAS available but a clinical description of normal muscle strength. 2 patients did not have a PGA available, but both had a CMAS of 52, no skin symptoms and low muscle enzymes. All patients “at diagnosis” were considered active, also because diagnoses of juvenile DM (JDM) were certain due to available clinical follow-up data. In the “AM” group 10 patients had missing PGA, 6 of which had evident muscle weakness (by CMAS or clinical description), with or without skin symptoms. The remaining 4 patients in this group had evident skin symptoms (CAT or clinical description). In the “R” group the median time between stopping immunosuppressive medication and sampling was 39.8 months, with a range of 4.7-106.3 months. *NR=not recorded, CMAS = childhood myositis assessment scale, PGA = physician’s global assessment, ANA = anti-nuclear antibodies, MSA = myositis-specific antibodies, IV = intravenous, CK = creatine kinase, AST = aspartate aminotransferase, ALT = alanine aminotransferase, LDH = lactate dehydrogenase, CRP = C-reactive protein, ESR = erythrocyte sedimentation rate, IU= international units.*

**Supplementary Table 3. Analysis of ROC curves for galectin-9 and CXCL10 in external and internal cross-sectional validation cohort.**

ROC curves for both the internal and external validation cohort, comparing galectin-9, CXCL10 and CK were constructed, assessing their discriminative power for active disease versus remission. Either all patients with active juvenile DM (JDM) and all patients in remission, regardless of treatment (“active versus remission”) or only patients on medication (“AM versus RM”) were analyzed. *AUC = area under the curve, AM = active on medication, RM = remissionon medication.*

**Supplementary Table 4. Patient characteristics of cohort with adult inflammatory myopathies and eosinophilic fasciitis.**

Patients were stratified based on disease activity, regardless of treatment. Of each patient, only one sample per category (active or remission) was included in the analysis. The internal validation cohort (supplementary table 2) served as the juvenile DM (JDM) cohort in this analysis, with exclusion of samples from the same patient falling within the same category (‘active’ or ‘remission’). Unless indicated otherwise, values represent: median (range). 6 out of 31 active patients had a CMAS>47, but were defined as active based on active skin disease and/or active arthritis, rendering an elevated PGA. 3 of these patients also had an elevated CK despite a normal CMAS. *HC = healthy adult control, NL = Netherlands (Dutch cohort), DM = adult dermatomyositis, NSM = adult non-specific myositis and overlap myositis, EF = eosinophilic fasciitis, SMA = spinal muscular atrophy, NR=not recorded, CMAS = childhood myositis assessment scale, VAS = visual analogue scale, mLoSSI = modified LS Skin Severity Index, HMFSE = Hammersmith Functional Motor Scale-Expanded, IV = intravenous, CK = creatine kinase, AST = aspartate Aminotransferase, ALT = alanineAminotransferase, LDH = lactate dehydrogenase, CRP = C-reactive protein, ESR = erythrocyte sedimentation rate.*

**Supplementary Table 5. Patient characteristics of cohort with systemic autoimmune diseases with involvement of the skin.**

Patients were stratified based on disease activity, regardless of treatment. Of each patient, only one sample per category (active or remission) was included in the analysis. Unless indicated otherwise, values represent: median (range). 2 out of 9 active patients had a CMAS>47, but were defined as active based on active skin disease. 1 of these patients also had an elevated CK despite a normal CMAS. *HC = healthy adult control, LoS = localized scleroderma, SLE = systemic lupus erythematosus, NR=not recorded, CMAS = childhood myositis assessment scale, VAS = visual analogue scale, mLoSSI = modified LS Skin Severity Index, SLEDAI = Systemic Lupus Erythematosus Disease Activity Index, IV = intravenous,CK = creatine kinase, AST = Aspartate Aminotransferase, ALT = Alanine Aminotransferase, LDH = lactatedehydrogenase, CRP = C-reactive protein, ESR = erythrocyte sedimentation rate, IU = international units.*

**Supplementary Table 6. Patient characteristics of longitudinal cohort.**

Patients were stratified into three groups based on the timing of a disease flare: Patients without flares (“no flare”, n=15), patients with a flare within the first year after diagnosis (“<12 months”, n=6) and patients with a flare after the first year (“>12 months”, n=7). Of the 7 patients with a flare after the first year, 4 patients had a sample available at diagnosis. These 4 are shown in more detail in figure 3 and supplementary figure 2. *CMAS =childhood myositis assessment scale, PGA = physician’s global assessment, CK = creatine kinase, AST = aspartate aminotransferase, ALT = alanine aminotransferase, LDH = lactate dehydrogenase, CRP = C-reactive protein, ESR = erythrocyte sedimentation rate, IU = international units.*

**Supplementary Table 7. Patient characteristics of dried blood spot cohort.**

Only juvenile DM (JDM) patients with active disease were included in this cohort, and compared to adult healthy controls. *HC= healthy adult control, NR=not recorded, CMAS = childhood myositis assessment scale, CK = creatine kinase, AST = aspartate aminotransferase, ALT = alanine aminotransferase, LDH = lactate dehydrogenase, CRP = C-reactive protein, ESR = erythrocyte sedimentation rate, IU = international units.*


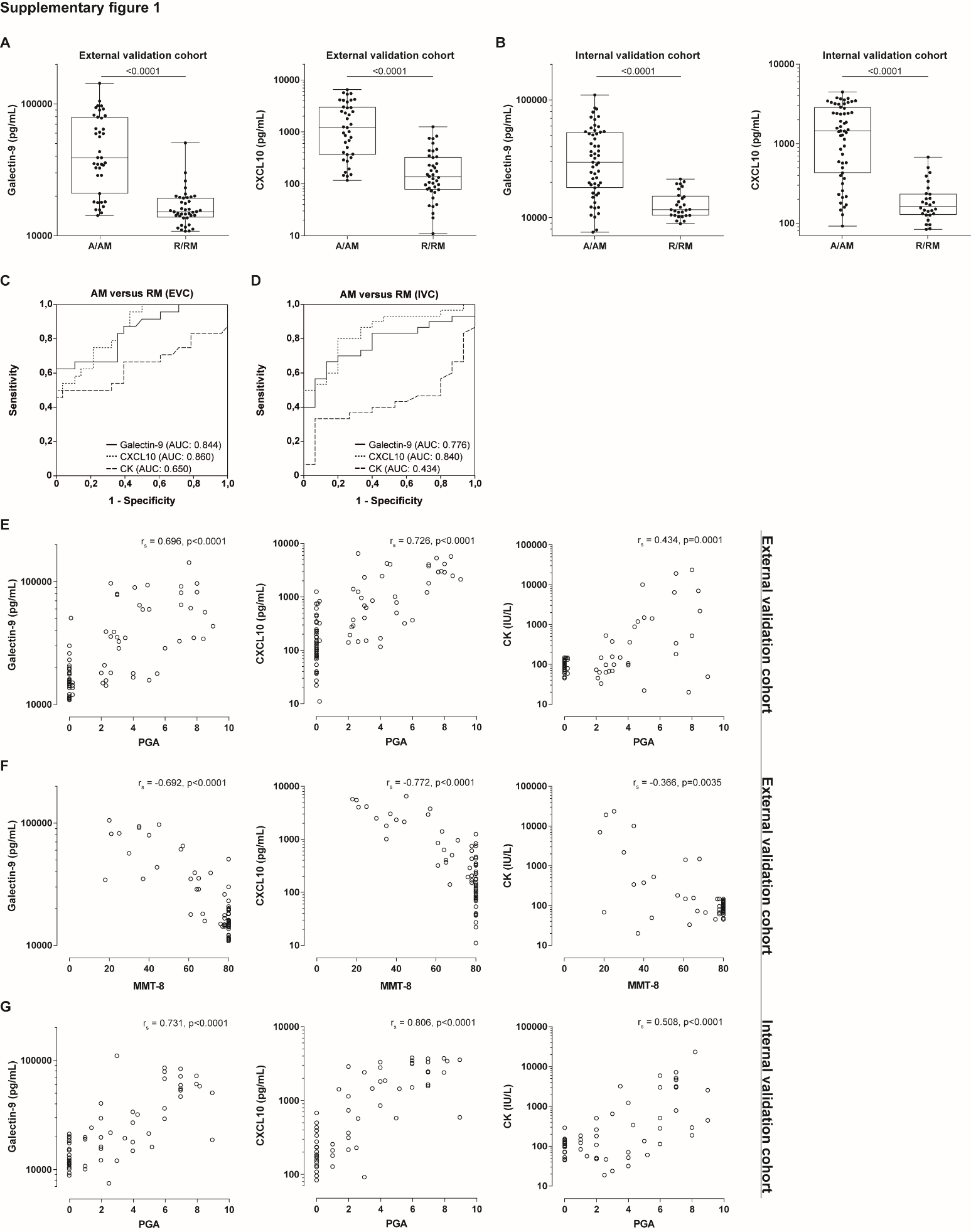
**Supplementary figure 1**

**
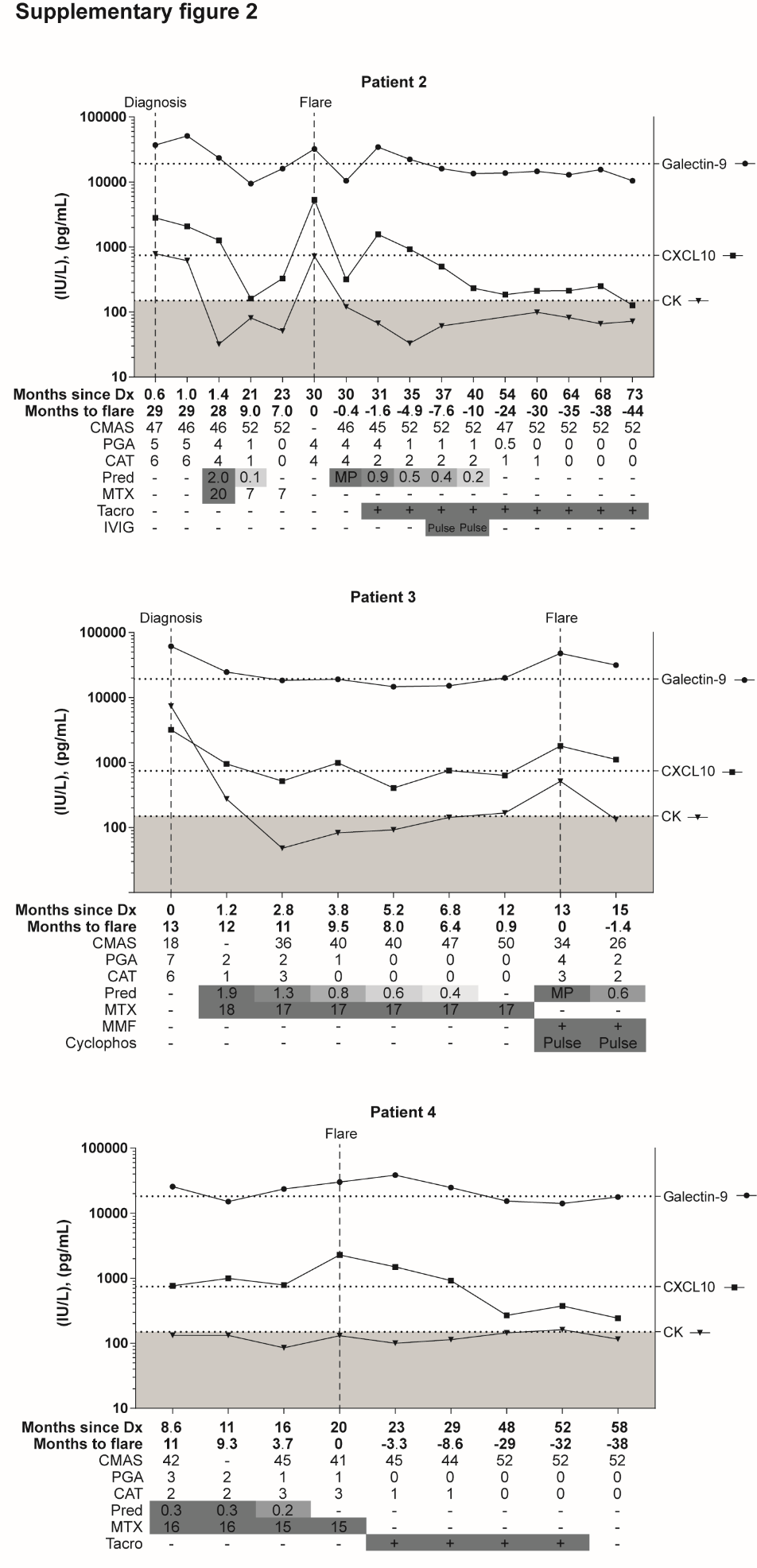
Supplementary figure 2**

**Supplementary Table 1**

|  | **External validation cohort (EVC) from London, United Kingdom** | | | |
| --- | --- | --- | --- | --- |
|  | **At diagnosis** | **Active on medication** | **Remission on medication** | **Remission off medication** |
|  | **A** | **AM** | **RM** | **R** |
|  | **(n=12)** | **(n=27)** | **(n=28)** | **(n=12)** |
| Age at diagnosis, years | 5 (2-15) | 6 (2-12) | 7 (2-14) | 6 (1-13) |
| Age at sampling, years | 5 (3-15) | 8 (3-16) | 10 (5-16) | 12 (6-17) |
| Sex, % female | 66.7 | 59.3 | 67.9 | 75.0 |
| Diagnosis to sampling, months | 0 (0-1) | 17 (0-107) | 34 (7-105) | 72 (42-127) |
| **Disease activity** |  |  |  |  |
| CMAS (0-52) | 7 (1-28) | 39 (3-44) | 52 (48-52) | 52 (50-52) |
|  | *NR=2* |  |  |  |
| PGA (0-10) | 7 (3-9) | 4 (2-8) | 0 (0-0) | 0 (0-0) |
|  | *NR=1* |  |  |  |
| MMT-8 score (0-80) | 35 (18-56) | 65 (25-78) | 80 (78-80) | 80 (78-80) |
|  | *NR=3* | *NR=11* |  |  |
| **Autoantibodies** |  |  |  |  |
| % of patients ANA positive | 58 | 63 | 46 | 42 |
| % of patients MSA positive | 88 | 93 | 77 | 67 |
|  | *NR=4* |  | *NR=2* |  |
| **Medication, % of patients** |  |  |  |  |
| Oral steroids | - | 73 | 7 | - |
| IV steroids | - | 36 | - | - |
| Methotrexate | - | 85 | 93 | - |
| Ciclosporin | - | 8 | - | - |
| Azathioprine | - | 4 | 14 | - |
| Cyclophosphamide | 8 | 25 | - | - |
| Hydroxychloroquine | - | 8 | 4 | - |
| IV immunoglobulins | 8 | 4 | 4 | - |
| Etanercept | - | - | 4 | - |
| Infliximab | - | 13 | 4 | - |
| None | 92 | - | - | 100 |
| **Muscle enzymes** |  |  |  |  |
| CK, IU/liter | 442 (49-19078) | 126 (20-23444) | 78 (45-147) | 87 (48-145) |
|  | *NR=2* | *NR=3* |  |  |
| AST, IU/liter | 153 (60-246) | 57 (27-876) | 49 (26-65) | 41 (38-43) |
|  | *NR=10* | *NR=20* | *NR=21* | *NR=10* |
| ALT, IU/liter | 85 (25-340) | 35 (10-482) | 23 (6-125) | 19 (6-39) |
|  | *NR=1* | *NR=4* |  |  |
| LDH, IU/liter | 1410 (686-4676) | 793 (404-5478) | 590 (405-829) | 589 (392-832) |
|  | *NR=1* | *NR=4* | *NR=1* | *NR=1* |
| **Inflammation markers** |  |  |  |  |
| CRP, mg/liter | 5 (5-9) | 7 (1-10) | 4 (3-63) | 5 (3-7) |
|  | *NR=2* | *NR=9* | *NR=5* |  |
| ESR, mm/hour | 36 (5-124) | 13 (1-80) | 6 (2-40) | 5 (2-26) |
|  | *NR=1* | *NR=1* |  | *NR=1* |

**Supplementary Table 2**

|  | **Internal validation cohort (IVC) from Utrecht, Netherlands** | | | |
| --- | --- | --- | --- | --- |
|  | **At diagnosis** | **Active on medication** | **Remission on medication** | **Remission off medication** |
|  | **A** | **AM** | **RM** | **R** |
|  | **(n=25)** | **(n=30)** | **(n=16)** | **(n=12)** |
| Age at diagnosis, years | 5 (2-16) | 6 (2-18) | 8 (3-16) | 7 (3-14) |
| Age at sampling, years | 5 (2-16) | 7 (2-18) | 9 (4-19) | 14 (8-25) |
| Sex, % female | 52.0 | 63.3 | 56.3 | 50.0 |
| Diagnosis to sampling, months | 0 (0-10) | 3 (0-80) | 18 (3-113) | 93 (37-191) |
| **Disease activity** |  |  |  |  |
| CMAS (0-52) | 21 (1-52) | 41 (10-52) | 52 (48-52) | 52 (48-52) |
|  | *NR=9* | *NR=9* | *NR=3* | *NR=1* |
| PGA (0-10) | 7 (2-9) | 2 (1-9) | 0 (0-0) | 0 (0-0) |
|  | *NR=6* | *NR=10* | *NR=3* | *NR=1* |
| **Autoantibodies** |  |  |  |  |
| % of patients ANA positive | 52 | 52 | 44 | 44 |
| % of patients MSA positive | 22 | 18 | 8 | 50 |
|  | *NR=7* | *NR=8* | *NR=3* | *NR=10* |
| **Medication, % of patients** |  |  |  |  |
| Oral steroids | - | 93 | 44 | - |
| Methotrexate | - | 80 | 56 | - |
| Cyclophosphamide | - | 10 | - | - |
| Hydroxychloroquine | - | 10 | 6 | - |
| IV immunoglobulins | - | 11 | - | - |
| Mycophenolate mofetil | - | 7 | 13 | - |
| Tacolimus | - | 3 | 13 | - |
| None | 100 | - | - | 100 |
| **Muscle enzymes** |  |  |  |  |
| CK, IU/liter | 813 (61-23908) | 83 (19-5292) | 112 (47-146) | 111 (45-293) |
|  |  |  | *NR=1* |  |
| AST, IU/liter | 186 (30-1343) | 34 (9-734) | 29 (19-40) | 24 (11-42) |
|  |  |  |  | *NR=1* |
| ALT, IU/liter | 63 (12-366) | 25 (11-1242) | 14 (8-28) | 18 (8-24) |
|  |  |  |  | *NR=1* |
| LDH, IU/liter | 658 (314-1541) | 281 (179-818) | 246 (160-347) | 205 (151-261) |
|  | *NR=3* | *NR=8* |  | *NR=2* |
| **Inflammation markers** |  |  |  |  |
| CRP, mg/liter | 1 (0-28) | 1 (0-8) | 1 (0-6) | 1 (1-9) |
|  | *NR=2* | *NR=3* | *NR=3* | *NR=3* |
| ESR, mm/hour | 16 (2-85) | 6 (2-24) | 6 (2-26) | 6 (2-15) |
|  | *NR=3* | *NR=4* | *NR=1* | *NR=1* |

**Supplementary Table 3**

|  |  | **Marker** | **AUC** | Standard Error | Asymptotic Significance | Asymptotic 95% Confidence Interval | |  |
| --- | --- | --- | --- | --- | --- | --- | --- | --- |
|  | Cohort |  |  |  |  | *Lower Bound* | *Upper Bound* | N |
| Active versus remission | EC | Galectin-9 | **0.894** | 0.037 | <0.0001 | *0.821* | *0.966* | n=34 for active n=40 for remission n=5 missing |
|  |  | CXCL10 | **0.877** | 0.039 | <0.0001 | *0.802* | *0.953* |  |
|  |  | CK | **0.682** | 0.070 | 0.0074 | *0.544* | *0.819* |  |
| AM versus RM | EC | Galectin-9 | **0.844** | 0.055 | <0.0001 | *0.738* | *0.951* | n=24 for active n=28 for remission n=3 missing |
|  |  | CXCL10 | **0.860** | 0.049 | <0.0001 | *0.764* | *0.956* |  |
|  |  | CK | **0.650** | 0.084 | 0.0638 | *0.485* | *0.815* |  |
| Active versus remission | IC | Galectin9 | **0.863** | 0.040 | <0.0001 | *0.785* | *0.941* | n=55 for active n=27 for remission n=1 missing |
|  |  | CXCL10 | **0.902** | 0.032 | <0.0001 | *0.838* | *0.965* |  |
|  |  | CK | **0.662** | 0.059 | 0.0170 | *0.547* | *0.778* |  |
| AM versus RM | IC | Galectin-9 | **0.776** | 0.069 | <0.0001 | *0.640* | *0.911* | n=30 for active n=15 for remission n=1 missing |
|  |  | CXCL10 | **0.840** | 0.060 | <0.0001 | *0.722* | *0.958* |  |
|  |  | CK | **0.434** | 0.086 | 0.4775 | *0.267* | *0.602* |  |

**Supplementary Table 4**

|  | **HC** | **JDM NL** | | **JDM Sing** | | **DM** | | **NSM** | | **EF** | | **SMA** |
| --- | --- | --- | --- | --- | --- | --- | --- | --- | --- | --- | --- | --- |
|  | **Utrecht, NL** | **Utrecht, NL** | | **Singapore** | | **Amsterdam, NL** | | **Amsterdam, NL** | | **Utrecht & Nijmegen, NL** | | **Utrecht, NL** |
|  |  | **Active disease** | **Remission** | **Active disease** | **Remission** | **Active disease** | **Remission** | **Active disease** | **Remission** | **Active disease** | **Remission** |  |
|  |  | **A/AM** | **RM/R** | **A/AM** | **RM/R** | **A/AM** | **RM/R** | **A/AM** | **RM/R** | **A/AM** | **RM/R** |  |
|  | **(n=22)** | **(n=31)** | **(n=27)** | **(n=8)** | **(n=6)** | **(n=28)** | **(n=8)** | **(n=9)** | **(n=5)** | **(n=11)** | **(n=7)** | **(n=43)** |
| Age at diagnosis, years | - | 6 (2-18) | 7 (3-16) | 7 (4-16) | 7 (4-11) | 47 (19-81) | 43 (18-63) | 45 (18-69) | 40 (34-43) | 59 (30-70) | 53 (41-64) | 2 (0-31) |
| Age at sampling, years | 31 (21-61) | 8 (2-18) | 13 (4-25) | 12 (7-16) | 11 (6-12) | 49 (19-81) | 54 (19-75) | 45 (18-69) | 46 (43-59) | 61 (34-69) | 57 (44-71) | 36 (2-71) |
| Sex, % female | 68.2 | 54.8 | 55.6 | 50.0 | 33.3 | 71.4 | 87.5 | 100.0 | 80.0 | 63.6 | 28.6 | 60.5 |
| Diagnosis to sampling, months | - | 0 (0-80) | 37 (3-191) | 0 (0-129) | 41 (2-105) | 0 (0-195) | 85 (10-236) | 0 (0-36) | 84 (12-296) | 13 (1-330) | 34 (23-167) | 383 (12-830) |
| **Disease activity** |  |  |  |  |  |  |  |  |  |  |  |  |
| Muscle weakness  (% of patients) | - | 81 | 0 | 50 | 0 | 68 | 0 | 88 | 20 | - | - | - |
|  |  |  |  |  |  |  |  | *NR=1* |  |  |  |  |
| (J)DM skin symptoms  (% of patients) | - | 90 | 0 | 100 | 0 | 100 | 13 | 0 | 0 | - | - | - |
|  |  |  |  |  |  |  |  |  |  |  |  |  |
| CMAS (0-52) | - | 28 (1-52) | 52 (48-52) | 46 (14-52) | 52 (52-52) | - | - | - | - | - | - | - |
|  |  | *NR=10* | *NR=4* |  |  |  |  |  |  |  |  |  |
| EF VAS activity (0-100) | - | - | - | - | - | - | - | - | - | 41 (7-64) | 2 (0-4) | - |
|  |  |  |  |  |  |  |  |  |  | *NR=2* | *NR=1* |  |
| EF activity (mLoSSI, 0-162) | *-* | - | - | - | - | - | - | - | - | 30 (14-52) | 8 (3-40) | *-* |
|  |  |  |  |  |  |  |  |  |  | *NR=2* | *NR=1* |  |
| SMA motor score  (HMFSE, 0-66) | - | - | - | - | - | - | - | - | - | - | - | 4 (0-60) |
|  |  |  |  |  |  |  |  |  |  |  |  | *NR=4* |
| **Medication, % of patients** |  |  |  |  |  |  |  |  |  |  |  |  |
| Oral steroids | - | 16 | 26 | 13 | 50 | 29 | 50 | 22 | 80 | 27 | 57 | - |
| Methotrexate | - | 16 | 33 | 25 | 67 | 4 | 25 | 11 | 40 | 9 | 71 | - |
| Azathioprine | - | - | - | - | - | - | 38 | - | - | - | - | - |
| Hydroxychloroquine | - | 3 | 4 | 38 | 67 | 4 | 13 | 22 | 20 | - | - | - |
| IV immunoglobulins | - | 3 | - | - | - | 7 | - | - | - | - | - | - |
| Tacrolimus | - | 3.2 | 4 | - | - | 4 | - | - | - | - | - | - |
| Mycophenolate Mofetil | - | 3.2 | 7 | 13 | - | - | - | - | - | - | - | - |
| Adalimumab | - | - | - | - | - | - | - | 11 | - | - | - | - |
| None | 100 | 81 | 44 | 50 | 17 | 68 | 25 | 44 | 0 | 73 | 14 | 100 |
| **Muscle enzymes** |  |  |  |  |  |  |  |  |  |  |  |  |
| CK, IU/liter | - | 658 (52-23908) | 109 (45-293) | 156 (84-1010) | 136 (101-184) | 268 (32-12338) | 87 (45-481) | 1518 (75-5700) | 94 (66-149) | 37 (35-39) | 18 (18-18) | - |
|  |  |  | *NR=1* |  |  | *NR=4* |  | *NR=1* |  | *NR=9* | *NR=6* |  |
| AST, IU/liter | - | 88 (12-1343) | 28 (11-42) | 33 (25-102) | 25 (18-31) | 46 (14-415) | 16 (13-37) | 100 (67-133) | 19 (15-23) | 24 (22-28) | - | - |
|  |  |  | *NR=1* |  |  | *NR=11* | *NR=3* | *NR=7* | *NR=1* | *NR=8* |  |  |
| ALT, IU/liter | - | 51 (11-1242) | 15 (8-28) | 20 (9-71) | 14 (11-24) | 33 (5-324) | 17 (10-59) | 60 (13-327) | 20 (18-24) | 23 (12-70) | 30 (20-62) | - |
|  |  |  | *NR=1* |  |  | *NR=8* |  | *NR=2* |  | *NR=6* | *NR=2* |  |
| LDH, IU/liter | - | 645 (189-1541) | 224 (151-347) | 318 (237-700) | 255 (224-276) | 313 (163-613) | 182 (163-200) | 170 (170-170) | 136 (136-136) | 215 (194-235) | - | - |
|  |  | *NR=3* | *NR=2* |  |  | *NR=11* | *NR=6* | *NR=8* | *NR=4* | *NR=9* |  |  |
| **Inflammation markers** |  |  |  |  |  |  |  |  |  |  |  |  |
| CRP, mg/liter | - | 1 (0-28) | 1 (0-9) | 1 (0-21) | 1 (0-1) | 5 (0-129) | 5 (2-8) | 4 (1-34) | 4 (3-14) | 10 (9-11) | 10 (2-12) | - |
|  |  | *NR=3* | *NR=6* |  |  | *NR=12* | *NR=4* | *NR=2* | *NR=2* | *NR=9* | *NR=4* |  |
| ESR, mm/hour | - | 14 (2-85) | 6 (2-26) | 13 (2-104) | 6 (2-16) | 11 (2-130) | 6 (3-12) | 31 (5-75) | 4 (3-14) | 13 (5-23) | 5 (2-102) | - |
|  |  | *NR=5* | *NR=2* |  |  | *NR=13* | *NR=4* | *NR=3* | *NR=2* | *NR=7* | *NR=2* |  |

**Supplementary Table 5**

|  | **HC** | **JDM** | | **LoS** | | **SLE** | |
| --- | --- | --- | --- | --- | --- | --- | --- |
|  | **Utrecht, NL** | **Utrecht, NL** | | **Utrecht & Nijmegen, NL** | | **Utrecht, NL** | |
|  |  | **Active disease** | **Remission** | **Active disease** | **Remission** | **Active disease** | **Remission** |
|  |  | **A/AM** | **RM/R** | **A/AM** | **RM/R** | **A/AM** | **RM/R** |
|  | **(n=12)** | **(n=9)** | **(n=7)** | **(n=8)** | **(n=7)** | **(n=19)** | **(n=17)** |
| Age at diagnosis, years |  | 12 (5-18) | 6 (5-14) | 10 (3-16) | 8 (1-13) | 19 (11-37) | 23 (11-29) |
| Age at sampling, years | 35 (25-57) | 12 (5-18) | 10 (7-17) | 12.5 (6-16) | 14 (7-16) | 33 (11-55) | 40 (12-56) |
| Sex, % female | 75 | 66.7 | 57.1 | 33.3 | 71.4 | 100.0 | 94.1 |
| Diagnosis to sampling, months |  | 0 (0-38) | 36 (5-77) | 10.5 (6-66) | 47 (10-180) | 131 (0-270) | 149 (7-472) |
| **Disease activity** |  |  |  |  |  |  |  |
| Muscle weakness  (% of patients) |  | 78 | 0 | - | - | - | - |
|  |  |  | *NR=1* |  |  |  |  |
| CMAS (0-52) |  | 35 (14-52) | 50 (48-52) | - | - | - | - |
|  |  | *NR=1* | *NR=2* |  |  |  |  |
| Juvenile DM skin symptoms  (% of patients) |  | 100 | 0 | - | - | - | - |
|  |  |  | *NR=1* |  |  |  |  |
| Morphea activity (mLoSSI, 0-162) |  | - | - | 8 (6-22) | 1 (0-5) | - | - |
|  |  |  |  | *NR=1* |  |  |  |
| Morphea VAS activity (0-100) |  | - | - | 19 (11-37) | 0 (0-5) | - | - |
|  |  |  |  | *NR=1* |  |  |  |
| SLEDAI (0-105) |  | - | - | - | - | 6 (4-26) | 2 (0-3) |
|  |  |  |  |  |  |  |  |
| LE skin disease  (% of patients) |  | *-* | *-* | *-* | *-* | 83 | 53 |
|  |  |  |  |  |  | *NR=1* |  |
| **Medication, % of patients** |  |  |  |  |  |  |  |
| Oral steroids |  | 22 | 71 | 17 | 14 | 68 | 35 |
| IV steroids |  | - | - | - | - | - | - |
| Methotrexate |  | 22 | 71 | 17 | 43 | - | - |
| Ciclosporin |  | - | - | - | - | - | - |
| Azathioprine |  | - | - | - | - | 42 | 6 |
| Cyclophosphamide |  | - | - | - | - | - | - |
| Hydroxychloroquine |  | - | - | - | - | 68 | 53 |
| IV immunoglobulins |  | - | - | - | - | - | - |
| Tacrolimus |  | - | 29 | - | - | - | - |
| Mycophenolate Mofetil |  | - | - | - | 29 | 32 | 29 |
| Etanercept |  | - | - | - | - | - | - |
| Infliximab |  | - | - | - | - | - | - |
| None |  | 67 | 14 | 83 | 29 | 11 | 29 |
| **Muscle enzymes** |  |  |  |  |  |  |  |
| CK, IU/liter |  | 1240 (52-3243) | 84 (45-370) | 33 (24-42) | 84 (59-183) | - | - |
|  |  |  |  | *NR=4* |  |  |  |
| AST, IU/liter |  | 112 (18-1343) | 22 (12-32) | 24 (24-24) | 27 (18-35) | - | - |
|  |  |  |  | *NR=5* |  |  |  |
| ALT, IU/liter |  | 85 (14-1242) | 16 (11-25) | 20 (16-23) | 19 (13-30) | 17 (10-123) | 17 (9-40) |
|  |  |  |  | *NR=1* | *NR=1* |  |  |
| LDH, IU/liter |  | 658 (213-1742) | 241 (160-255) | - | - | - | - |
| **Inflammation markers** |  |  |  |  |  |  |  |
| CRP, mg/liter |  | 2 (0-7) | 1 (0-5) | 5 (5-5) | 5 (1-9) | 1 (1-13) | 2 (1-18) |
|  |  |  |  | *NR=3* |  | *NR=8* | *NR=8* |
| ESR, mm/hour |  | 9 (5-33) | 7 (2-14) | 5 (2-8) | 2 (0-6) | 16 (2-82) | 11 (2-79) |
|  |  |  |  | *NR=2* |  | *NR=2* | *NR=2* |

**Supplementary Table 6**

|  | **JDM** | | |
| --- | --- | --- | --- |
|  | **Utrecht, NL** | | |
|  | **No flare** | **Flare < 12 months** | **Flare >12 months** |
|  | **(n=15)** | **(n=6)** | **(n=7)** |
| Age at diagnosis, years | 6 (3-15) | 9 (2-12) | 8 (3-15) |
| Sex, % female | 46.7 | 66.7 | 42.9 |
| Diagnosis to sampling, months | 0 (0-3) | 0 (0-0) | 0 (0-9) |
| Onset of disease to flare, months | - | 4 (3-10) | 30 (13-53) |
| Follow-up time, years | 2 (1-9) | 1 (1-10) | 3 (1-6) |
|  |  |  |  |
| **Disease activity at diagnosis** |  |  |  |
| CMAS (0-52) | 28 (1-37) | 14 (6-14) | 34 (18-47) |
|  |  | *NR=3* | *NR=1* |
| PGA (0-10) | *4 (2-9)* | *6 (5-7)* | *5 (3-7)* |
|  | *NR=2* |  |  |
| JDM skin symptoms (% of patients) | 100 | 100 | 100 |
|  |  |  |  |
| **Muscle enzymes at diagnosis** |  |  |  |
| CK, IU/liter | 510 (78-4687) | 683 (114-6038) | 193 (98-7334) |
|  |  |  |  |
| AST, IU/liter | 121 (35-1482) | 267 (63-940) | 57 (24-400) |
|  | *NR=1* |  |  |
| ALT, IU/liter | 76 (14-1242) | 99 (31-320) | 29 (16-77) |
|  | *NR=1* |  |  |
| LDH, IU/liter | 678 (316-958) | 754 (564-1292) | 428 (323-1028) |
|  | *NR=1* |  |  |
| **Inflammation markers at diagnosis** |  |  |  |
| CRP, mg/liter | 2 (0-28) | 2 (1-4) | 2 (1-7) |
|  | *NR=3* |  |  |
| ESR, mm/hour | 15 (2-121) | 28 (9-45) | 10 (5-40) |
|  | *NR=3* |  |  |

**Supplementary Table 7**

|  | **JDM** | **HC** |
| --- | --- | --- |
|  | **Utrecht, NL** | **Utrecht, NL** |
|  | **Active disease** |  |
|  | **A/AM** |  |
|  | **(n=10)** | **(n=12)** |
| Age at diagnosis, years | 4 (3-6) | - |
| Age at sampling, years | 4 (3-6) | 34 (21-61) |
| Sex, % female | 50.0 | 66.7 |
| Diagnosis to sampling, months | 0 (0-5) | - |
|  |  |  |
| **Disease activity** |  |  |
| CMAS (0-52) | 34 (1-42) | - |
|  |  |  |
| JDM skin symptoms (% of patients) | 75 | - |
|  |  |  |
| **Medication, % of patients** |  |  |
| Oral steroids | 50 | - |
| Methotrexate | 50 | - |
| Hydroxychloroquine | 10 | *-* |
| None | 50 | 100 |
|  |  |  |
| **Muscle enzymes** |  |  |
| CK, IU/liter | 99 (47-7334) | - |
|  |  |  |
| AST, IU/liter | 38 (22-400) | - |
|  |  |  |
| ALT, IU/liter | 25 (12-121) | - |
|  |  |  |
| LDH, IU/liter | 370 (284-1028) | - |
|  | *NR=3* |  |
| **Inflammation markers** |  |  |
| CRP, mg/liter | 1 (1-205) | - |
|  |  |  |
| ESR, mm/hour | 12 (2-85) | - |
|  |  |  |
